# Risk Factors Associated With Early-Onset Colorectal Neoplasm in Chinese Youth: A Prospective Population-Based Study

**DOI:** 10.3389/fonc.2021.702322

**Published:** 2021-10-08

**Authors:** Jie Shen, Yiling Wu, Miao Mo, Xiaoshuang Feng, Changming Zhou, Zezhou Wang, Guoxiang Cai, Ying Zheng

**Affiliations:** ^1^ Department of Cancer Prevention, Fudan University Shanghai Cancer Center, Shanghai, China; ^2^ Department of Oncology, Shanghai Medical College, Fudan University, Shanghai, China; ^3^ Department of Noninfectious Chronic Disease Control and Prevention, Shanghai Songjiang District Center for Disease Control and Prevention, Shanghai, China; ^4^ Department of Colorectal Surgery, Fudan University Shanghai Cancer Center, Shanghai, China

**Keywords:** risk factors, prospective population-based study, colorectal cancer, early-onset, screening

## Abstract

Evidence of the risk factors associated with early-onset colorectal neoplasm from prospective population-based studies is limited. We enrolled 17,293 participants younger than 50 years from the Shanghai colorectal cancer (CRC) screening program cohort. Face-to-face interviews were performed by trained primary care physicians using a standardized questionnaire to collect the information on potential risk factors at baseline entry. Furthermore, 124 cases of early-onset colorectal neoplasm, including six CRC cases and 118 colorectal adenoma (CRA) cases, were detected between 2012 and 2016. Multivariable logistic regression models and restricted cubic spline (RCS) were used to evaluate the risk factors associated with early-onset colorectal neoplasm. We found that sex, body mass index (BMI), and family history of CRC were associated with the early onset of colorectal neoplasm. The RCS model showed a positive dose–response and linear association between BMI and risk of early-onset colorectal neoplasm among young participants (p-overall = 0.19, p-nonlinear = 0.97). The findings indicated that it was beneficial for normal people younger than 50 years to start opportunistic CRC screening. As for those at high risk, increased surveillance is strongly recommended. Further close follow-up is required for research on the underlying causes of early-onset CRC.

## Highlights

In a population-based screening cohort with 17,293 participants younger than 50 years, 124 cases of early-onset colorectal neoplasm, including six cancer cases and 118 adenomas cases, were detected from 2012 to 2016. Sex, body mass index (BMI), and family history of colorectal cancer (CRC) were associated with early-onset colorectal neoplasm. Our results provided evidence for the efficacy of screening in Chinese youth and the risk factors associated with early-onset CRC.

## Introduction

The burden of colorectal cancer (CRC) incidence and mortality is rapidly growing worldwide. According to GLOBOCAN 2020 (1), more than 1.9 million new CRC cases and 935,000 deaths were estimated to occur, representing about one in 10 cancer cases and deaths. CRC incidence and mortality rates have declined substantially in the United States (US) over the past four decades due to CRC screening and improvements in treatment (2). However, the decrease in incidence and mortality occurred mostly among adults older than 50 years. In contrast, the incidence of CRC kept increasing and nearly doubled in younger adults since the 1990s (3, 4). Approximately one in 10 new patients with CRC in the US is younger than 50 years, and this early-onset CRC now accounts for 10%-12% of all new CRC diagnoses (5). In China, a steady increase in CRC incidence was observed over the past three decades, with the most pronounced increase in the youth aged 15-49 years (6). Therefore, the increasing incidence of CRC among younger individuals has become a global trend; however, the reason remains unclear.

Little is known about the mechanisms and factors responsible for the development of this early-onset CRC. Evidence suggested that early-onset CRC had a different clinical and molecular profile compared with traditional late-onset CRC (7). Epidemiological observation showed that early-onset CRC was characterized by a more advanced stage at diagnosis, poorer cell differentiation, higher prevalence of signet ring cell histology, and left colon-sided location of the primary tumor (8, 9). Some risk factors, such as obesity, smoking, alcohol, and consumption of red or processed meat, have been demonstrated to be independent risk factors associated with late-onset CRC (10–14). However, whether these environmental exposures and lifestyle factors play similarly important roles in early-onset CRC is poorly understood. The consensus regarding lifestyle-related and environmental risk factors associated with early-onset CRC requires more population-based studies.

In addition, the American Cancer Society (ACS) recently recommended lowering the beginning age of CRC screening to 45 years considering the prolonged trends in the increasing CRC disease burden among persons younger than 50 years (2, 15, 16) and based on the modeling analyses showing efficient strategies for CRC screening starting at the age of 45 years (17, 18). The implementation of widening the screening age gap and lowering the beginning age of screening has generated controversies. The present evidence for the efficacy of screening is limited to adults older than 50 years (19). The next question is whether CRC screening, which has demonstrated great benefits for older adults, is of great value to younger people.

Therefore, the present study used data from a large community-based CRC screening program cohort to provide evidence for the efficacy of screening in Chinese youth and further identify the risk factors associated with early-onset CRC.

## Materials and Methods

### Study Design and Population

The data were obtained from local Songjiang District community residents in the city of Shanghai, east of China. Songjiang District, located in the northwest suburban part of Shanghai, with an area of 464.2 km^2^ and a population of 1,568,000 residents in 2015, represents characteristics of the typical urban–rural integration area of the Chinese population. The Songjiang local CRC screening cohort was initially established in 2012 and covered almost all native residents by the end of 2016.

The details of the design and procedures of the Shanghai CRC screening program were described previously (20). In brief, local residents aged 50-74 years with no history of CRC were invited to participate in screening. The screening involves two steps: initial screening [including fecal immunochemical test (FIT) and risk assessment], followed by diagnostic testing of colonoscopy for individuals with positive results. Residents aged 50-74 years were the target population, and the fees were covered by the local government and personal health insurance account. Residents younger than 50 years were also welcomed to participate in screening but at their own expense.

Participants who met the following criteria were enrolled in this study: (1) asymptomatic residents younger than 50 years; (2) individuals who signed informed consent; and (3) individuals completing the baseline risk interview.

**Figure 1:**
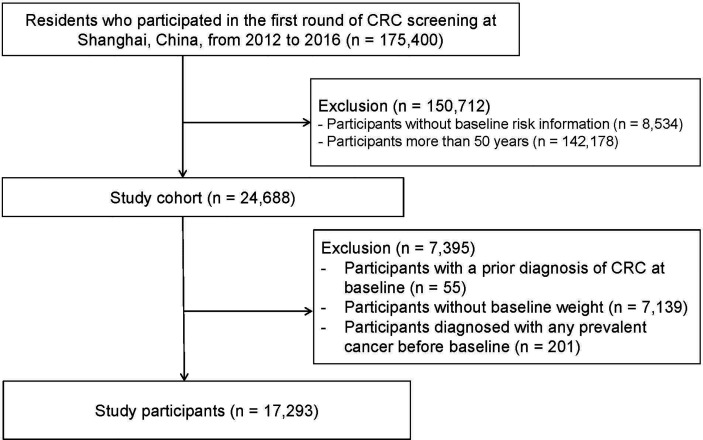
Flow diagram for selection of study cohort and participants.

We excluded individuals with a prior diagnosis of CRC at baseline (*n* = 55). Participants who did not report baseline weight were also excluded (*n* = 7,139). In the case of the potential survival bias, individuals diagnosed with any prevalent cancer before baseline were also excluded (*n* = 201) (**Figure 1**).

### Ascertainment of Colorectal Neoplasm

We defined all colorectal neoplasms, including colorectal adenomas (CRAs), carcinoma *in situ*, and CRC, as our primary end point. Participants were followed up from 2012 until the occurrence of cancer, death, or December 31, 2016. Participants with cancer were tracked through CRC screening results and annual searches of the Shanghai cancer registry system. CRC cases were defined as colorectal adenocarcinoma and confirmed by hospital medical records or pathology reports of colonoscopy. CRA cases were defined as histologically classified tubular adenomas, mixed adenomas, and villous adenomas and confirmed by the histopathological report of colonoscopy. The outcome information was further confirmed by a medical record review performed by clinical experts.

### Exposure Assessment

Face-to-face interviews were performed by trained primary care physicians for all participants using a standardized questionnaire to collect the information on potential risk factors at baseline entry. The questionnaire comprised 30 questions, including clinical anorectal symptoms; related colorectal diseases such as polyps and appendicitis; CRC family history; personal history of chronic diseases such as diabetes, hypertension, and hyperlipidemia; personal history of cancer history; and lifestyle factors such as smoking, alcohol drinking, and so forth. The educational level was classified as illiterate/primary school, middle school, and college/university. The marital status was classified as married, unmarried, divorced, and widowed. The smoking status was classified as current, ever smokers, and nonsmokers. The alcohol drinking status was classified as light drinkers (drinking less than once per month for more than 6 months), drinkers (drinking more than once per month for more than 6 months), and nondrinkers. Body weight and height were measured while participants were wearing light clothes without shoes, and the body mass index (BMI) was calculated using the following equation: BMI = weight (kg)/height (m^2^).

### Statistical Analysis

Numerical data were shown as means and standard deviation (SD) and compared with independent-sample *t* tests. Categorical variables were described as percentages and compared using the chi-square test. Binary logistic regression models adjusted for suspected confounders were used to calculate odds ratio (OR) and 95% confidence interval (CI) for risk factors and early-onset colorectal neoplasm, with adjustments for sex (male/female), educational level (illiterate/primary school, middle school, and college/university), marital status (married, unmarried, divorced, and widowed), BMI (continuous variable), family history of CRC (yes/no), personal history of diabetes (yes/no), frequency of smoking (nonsmoker, ever smoker, and current smoker), and frequency of alcohol drinking [nondrinker, light drinker (<1 time per month for more than 6 months), and drinker (≥1 time per month for more than 6 months)]. The restricted cubic spline (RCS) analysis was used in the multivariate logistic regression to explore the association between continuous BMI and early-onset colorectal neoplasm in the study.

We conducted the sensitivity analysis of risk factors for early-onset colorectal neoplasm by excluding the participants who had a positive history of chronic enteritis (including a history of inflammatory bowel disease (IBD), Crohn’s disease, and ulcerative colitis) (*n* = 461) and history of polyps (*n* = 280), which might lead to the detection of symptom bias.

The data management and all analyses were conducted using SAS statistical software, version 9.4 (SAS Institute). A p-value <0.05 indicated a statistically significant difference.

## Results

### Characteristics of the Screening Cohort Aged Younger Than 50 Years

Among the 17,293 participants screened, 43.23% were men. The mean age was 47.82 years (SD 2.60 years), almost all the participants (96.95%) were married, and the major education level was middle school or lower (95.75%). Very few (3.18%) had a family history of CRC. Among 2,950 FIT-positive participants, 874 (29.63%) underwent colonoscopy. The characteristics of the screening cohort aged younger than 50 years are shown in **Table 1**.

**Table 1:** Characteristics of screening cohort younger than 50 years.

	Screening negative (*N* = 17,169)	CRA (*N* = 118)	Cancer (*N* = 6)	Total screening participants (*N* = 17,293)
**Age (year), mean(SD)**	47.82 (2.60)	48.09 (1.59)	47.17 (4.49)	47.82 (2.60)
**Height (cm), mean(SD)**	163.83 (13.90)	166.06 (7.17)	164.67(7.53)	163.84 (13.86)
**Sex**				
Male	7,395 (43.07)	78 (66.10)	3 (50.00)	7,476 (43.23)
**BMI (kg/m^2^), mean(SD)**	23.81 (2.85)	24.57 (2.91)	24.17 (3.80)	23.81 (2.85)
**Marital status**				
Married	16,612 (96.94)	117 (99.15)	5 (83.33)	16,734 (96.95)
Unmarried	127 (0.74)	0 (0.00)	0 (0.00)	127 (0.73)
Divorced	271 (1.58)	1 (0.85)	0 (0.00)	272 (1.58)
Widowed	127 (0.74)	0 (0.00)	1 (16.67)	128 (0.74)
**Education**				
Illiterate/Primary school	4,014 (23.38)	19 (16.10)	3 (50.00)	4,036 (23.34)
Middle school	12,424 (72.36)	96 (81.36)	1 (16.67)	12,521 (72.40)
College/University	731 (4.26)	3 (2.54)	2 (33.33)	736 (4.26)
**Family history of colorectal cancer**	532 (3.11)	16 (13.56)	1 (16.67)	549 (3.18)
**History of diabetes**	776 (4.52)	5 (4.24)	0 (0.00)	781 (4.52)
**Smoking**				
Nonsmoker	11,842 (68.98)	57 (48.31)	5 (83.33)	11,904 (68.84)
Former smoker	341 (1.99)	3 (2.54)	0 (0.00)	344 (1.99)
Current smoker	4,985 (29.03)	58 (49.15)	1 (16.67)	5,044 (29.17)
**Alcohol** ^a^				
Nondrinker	13,756 (80.16)	80 (67.80)	4 (66.67)	13,840 (80.07)
Light drinker	474 (2.76)	2 (1.69)	0 (0.00)	476 (2.75)
Drinker	2,931 (17.08)	36 (30.51)	2 (33.33)	2,969 (17.18)
**FIT**				
Positive	2,826 (16.45)	118 (100.00)	6 (100.00)	2,950 (17.06)
**Colonoscopy compliance** ^b^	750 (26.54)	118 (100.00)	6 (100.00)	874 (29.63)

BMI, body mass index; CRA, colorectal adenoma; FIT, fecal immunochemical test; SD, standard deviation.

a Alcohol drinking was classified as nondrinker, light drinker (<1 time per month for more than 6 months), and drinker (≥1 time per month for more than 6 months).

b Colonoscopy compliance means the participation rate of colonoscopy among those with positive FIT results.

By December 31, 2016, a total of 124 patients with colorectal neoplasm were newly diagnosed, including six cases of early-onset CRC (median age at diagnosis, 49 years) and 118 CRA cases (median age at diagnosis, 48 years). The overall detection rate for CRAs and cancer was 682.36 per 100,000 and 34.69 per 100,000, respectively. Participants with CRC and CRA were taller and had a higher proportion of males and a positive family history of CRC. Moreover, participants with CRC and CRA were more likely to have higher BMI and more likely to smoke and drink alcohol.

### Risk Factors for Early-Onset Colorectal Neoplasm

**Figure 2:**
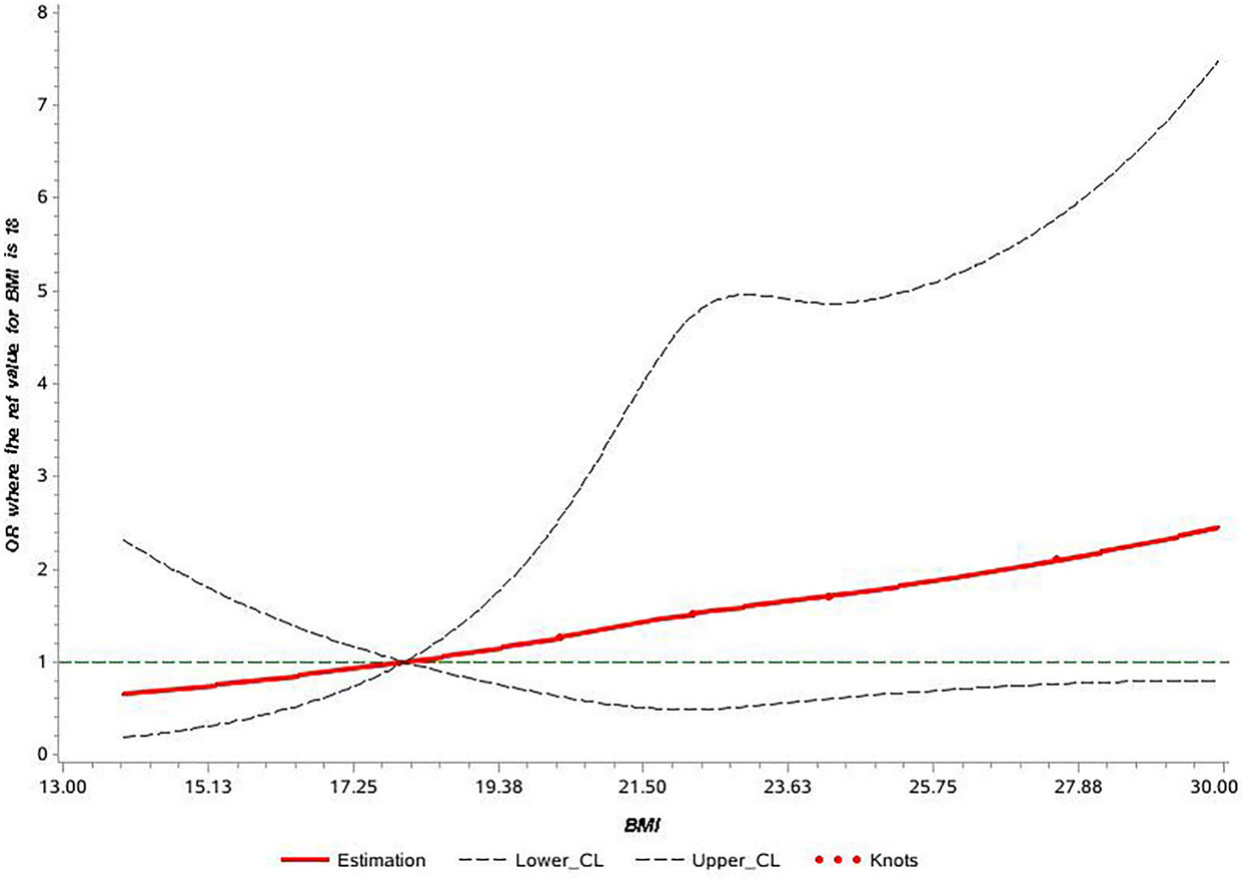
Cubic spline graph of the adjusted odds ratio (OR) (shown in solid line) and 95% CI (the dotted lines) for the association between body mass index (BMI) and risk of early-onset colorectal neoplasm in Shanghai colorectal cancer (CRC) screening cohort, 2012-2016. Knots: 18, 24, and 28 of BMI (kg/m^2^); Referent: 18 kg/m^2^.

The univariate analyses showed that sex, BMI, family history of CRC, smoking, and alcohol drinking were associated with early-onset colorectal neoplasm (**Table 2**). After adjusting for suspected confounders, the multivariable regression analysis indicated that sex, family history, and BMI were important risk factors associated with early-onset colorectal neoplasm. The male sex was associated with a 94% (95% CI: 1.12-3.36) higher risk of early-onset colorectal neoplasm. Participants with a positive family history of CRC had a five times higher risk of early-onset colorectal neoplasm (95% CI: 3.07-8.72). BMI was independently associated with a 7% increased risk of early-onset colorectal neoplasm (95% CI: 1.01-1.14). The RCS model showed a positive dose-response and linear relationship between the level of BMI and the risk of early-onset colorectal neoplasm among young participants (p-overall = 0.19, p-nonlinear = 0.97) (**Figure 2**).

**Table 2:** Risk factors associated with early-onset colorectal neoplasm.

	Crude OR (95% CI)	Multivariable-adjusted OR^a^ (95% CI)
**Male**	2.49 (1.72-3.61)	1.94 (1.12-3.36)
**BMI**	1.09 (1.03-1.15)	1.07 (1.01-1.14)
**Family history of colorectal cancer**	4.96 (2.95-8.33)	5.17 (3.07-8.72)
**Smoking**		
Nonsmoker	1	1
Ever smoker	1.68 (0.52-5.38)	0.92 (0.27-3.12)
Current smoker	2.26 (1.58-3.23)	1.27 (0.75-2.15)
**Alcohol** ^b^		
Nondrinker	1	1
Light drinker	0.69 (0.17-2.82)	0.42 (0.10-1.74)
Drinker	2.12 (1.44-3.12)	1.20 (0.76-1.89)

BMI, body mass index; CI, confidence interval; OR, odds ratio.

a Adjusted for educational level (illiterate/primary school, middle school, and college/university), marital status (married, unmarried, divorced, and widowed), and personal history of diabetes (yes/no).

b Alcohol drinking was classified as nondrinker, light drinker (<1 time per month for more than 6 months), and drinker (≥1 time per month for more than 6 months).

### Sensitivity Analysis

In the sensitivity analysis, the ORs remained robust when excluding the participants who had a positive history of chronic enteritis (*n* = 461) of sex (OR = 1.87, 95% CI: 1.06-3.29), BMI (OR = 1.07, 95% CI: 1.00-1.14), and family history of CRC (OR = 4.91, 95% CI: 2.83-8.54). In addition, excluding participants who had a positive history of polyps (*n* = 280), still a positive association was found between risk of the early-onset colorectal neoplasm and sex (OR = 2.04, 95% CI: 1.14-3.66), BMI (OR = 1.08, 95% CI: 1.01-1.15), and family history of CRC (OR = 5.26, 95% CI: 3.02-9.16) (**Table 3**).

**Table 3:** Sensitivity analyses of the risk factors associated with early-onset colorectal neoplasm.

	Male	BMI	Family history of CRC
**Analysis after excluding participants with chronic enteritis history**			
**Crude OR (95% CI)**	2.33 (1.59-3.41)	1.09 (1.02-1.15)	4.84 (2.80-8.39)
OR^a^	1.87 (1.06-3.30)	1.07 (1.00-1.14)	4.92 (2.83-8.56)
**Analysis after excluding participants with chronic polyps history**			
**Crude OR (95% CI)**	2.66 (1.79-3.95)	1.10 (1.04-1.17)	5.01 (2.89-8.70)
OR^a^	2.04 (1.14-3.66)	1.08 (1.01-1.15)	5.26 (3.02-9.16)

BMI, body mass index; CI, confidence interval; OR, odds ratio; CRC, colorectal cancer.

a Adjusted for educational level (illiterate/primary school, middle school, and college/university), marital status (married, unmarried, divorced, and widowed), smoking status (current smoker, ever smoker, and nonsmoker), personal history of diabetes (yes/no), alcohol drinking status (light drinker: drinking <1 time per month for more than 6 months, drinker: drinking more than once per month for more than 6 months, and nondrinker).

## Discussion

This large prospective cohort study on Chinese youth included 17,293 local residents younger than 50 years who participated in the CRC screening program in Shanghai. Furthermore, 124 cases of early-onset colorectal neoplasm, including six CRC cases and 118 cases of CRA, were detected. Furthermore, sex, BMI, and family history of CRC were found to be significantly associated with the risk of early-onset colorectal neoplasm. This study was among the first prospective studies to provide evidence for the screening benefit in the youth and investigate prospectively the risk factors associated with early-onset colorectal neoplasm among the youth in mainland China.

In our study, six CRC cases and 118 CRA cases were screened out of 17,293 participants younger than 50 years with the detection rate of 34.70/100,000 and 682.36/100,000, respectively. The detection rate was relatively lower than that in participants aged 50-74 years in Shanghai in 2013 (201.35/100,000 for CRC and 823.69/100,000 for CRA) (20) and even much lower than that in participants aged 65-75 years in Dutch in 2014 (4.7/1,000 for CRC and 23/1,000 for advanced adenomas) (21). This was in line with the increasing trends in CRC incidence with age in Shanghai (22). Moreover, a low detection rate might also be related to the low participation rate. A Japanese national survey on the implementation of CRC screening showed that the participation rates of individuals aged 40-49 years was only 15.69%, much lower than that of individuals in their 50s, 60s, and 70s (23). Besides, a low participation rate in our study could be partly due to the fact that the young people were not the target group of CRC screening. In terms of screening age, the recommendation of starting age in current guidelines from all major organizations was at the age of 50 years (19). However, lowering the beginning age to 45 years might provide unexpected benefits for those aged between 45 and 50 years. Concerning the great loss in life-years when young people develop CRC, it might be worth starting opportunistic screening at the age younger than 50 years, especially in those with increased CRC risk. Regarding the organized population-based CRC screening program, more evidence is needed for the recommendation of lowering the beginning age. Geographical variations in CRC incidence, economic resources, healthcare structure, and infrastructure to support screening, such as the ability to identify the target population at risk and cancer registry availability, should also be taken into account (19).

Our finding that the BMI level was associated with a significantly increased risk of early-onset colorectal neoplasm in this study was in line with the observations in existing studies. A case-control study in Iran youth showed that the incidence of overweight and/or obesity (BMI >25 kg/m^2^) was significantly higher in adenoma-positive patients compared with controls (49.9% and 0.9%, respectively, p = 0.04) (11). A retrospective cross-sectional study with 37,483,140 average-risk adults and 16,090 sporadic early-onset CRC in the US showed that obesity (BMI ≥30 kg/m^2^) was an independent risk factor for early-onset CRC at the age of 20-39 years (men: OR = 1.92, 95% CI: 1.85-1.99; women: OR = 2.22, 95% CI: 1.84-2.43) and the age of 40-49 years (men: OR = 1.96, 95% CI: 1.87-2.06; women: OR = 1.49, 95% CI: 1.41-1.57) (24). Another retrospective analysis of 72,356 asymptomatic persons aged less than 40 years in Korea found that obesity (BMI ≥25.0 kg/m^2^) might increase the risk of early-onset adenomas and advanced early-onset colorectal neoplasm (multivariate OR = 1.26, 95% CI: 1.19–1.34; OR = 1.33, 95% CI: 1.11–1.61) (25). The prospective Nurses’ Health Study II cohort in the US found that the risk of early-onset CRC was 37% higher in overweight women (BMI = 25.0-29.9 kg/m^2^) and 93% higher in obese women (BMI ≥30.0 kg/m^2^) (HR = 1.37, 95% CI: 0.81-2.30; HR = 1.93, 95% CI: 1.15-3.25) compared with women with a normal BMI of 18.5-22.9 (26). It has been widely reported that BMI is associated with late-onset CRC. We might need to study BMI across a lifetime, so as to provide more information on the association with early-onset CRC and identify periods of exposure that have the greatest effects on risks.

In our present study, one out of six early-onset CRC cases had a positive family history, which was lower than that in the US where approximately four of 10 patients with newly diagnosed early-onset CRC had a positive family history of the disease (5). The youth was likely to have a less positive rate of family history. A retrospective population-level cohort study in Western Austria indicated that individuals aged 50–69 years were more likely to have a first-degree relative with CRC (9.7%) compared with those aged 18–49 years (2.9%) (27). In agreement with previous reports that positive family history had a very close association with CRC or colorectal neoplasm incidence (28, 29), our study also showed the significant association between family history and the risk of early-onset colorectal neoplasm (OR = 5.17, 95% CI: 3.07-8.72).

In this study, we found that the risk of early-onset colorectal neoplasm was much higher in men than in women, which was in line with the observations in late-onset CRC reported previously (30, 31). Several possible mechanisms might explain higher risks in men. Sex hormonal factors and metabolic syndrome (MS), as well as complex interactions between these influences, might be responsible for the difference. Studies indicated that the risk of CRC with MS was either observed only in men (OR = 1.86, 95% CI: 1.21-2.86; OR = 1.13, 95% CI: 0.66-1.93 for men and women, respectively) (32) or stronger in men (OR for CRC = 1.78 and 1.16 for men and women, respectively) (33). Similar findings were reported for CRA (OR = 1.44, 95% CI: 1.16-1.80; OR = 1.04, 95% CI: 0.74-1.46 for men and women, respectively) (34).

The major limitation of our study was the relatively small sample size and small number of cancer cases, which was mainly due to the low participation rate and short follow-up time, limiting the cancer-specific analysis and detailed clinical, pathological, or even genetic analysis. A longer follow-up time is required for further research on potential specific risk factors associated with early-onset CRC. The second limitation was the possible selection bias due to the health worker effect. Youth aged less than 50 years who came to participate in this screening were keen to care about personal health, which might have underestimated our result. Third, not all participants in this study underwent colonoscopy, and a certain number of CRC or CRA cases might have been misclassified as the screening negative participants, leading to misclassification bias. However, the misclassification of positive cases as negative was to some extent a dilution of the results, making the present results relatively conservative. The association between risk factors and colorectal neoplasm would be even greater. Furthermore, more in-depth and further extensive research is required due to the non-availability of the data for certain significant anthropometric and clinicopathological features/parameters (nutritional, lifestyle, and potential risk factors) and prevalence of chronic conditions, as well as the limited analysis.

Nevertheless, the use of a population-based cohort of younger people significantly increased the strength of this study. Moreover, prospective collection of data and assessments of weight, height, and other risk factors could minimize the influence of confounding bias, recall bias, measurement errors, and information errors.

In summary, our study focused on the implementation of CRC screening in Chinese young people younger than 50 years and provided convincing evidence that sex, BMI, and family history of CRC were independent risk factors for early-onset colorectal neoplasm in Chinese youth. These findings indicated that it might be beneficial for normal people to start opportunistic CRC screening at the age of less than 50 years. A personalized screening approach with increased surveillances is strongly recommended for people at a high risk such as those with a positive family history or higher BMI. Further close follow-up is required for research on the underlying causes and mechanisms of early-onset CRC, especially on genetic mechanisms. Genetic testing of early-onset CRC patients could guide precision treatment and help in early detection of at-risk people. The discovery of genetic and molecular mechanisms will provide additional information on further new treatment approaches.

## Data Availability Statement

The raw data supporting the conclusions of this article will be made available by the corresponding author upon reasonable request. 

## Ethics Statement

All participants provided written informed consent, and research ethics approval was waived by the institutional research committee of Shanghai Municipal Center of Disease Control and Prevention because it was a public program covering all citizens at a certain range of ages in Shanghai.

## Author Contributions

JS, YW, GC, and YZ had the idea for this study. YW and YZ contributed to the data collection and supervised the acquisition of the data. MM and XF provided statistical advice. CZ and ZW undertook the statistical analysis. All authors contributed to interpretation of the results. JS and YW wrote the article. GC and YZ revised the manuscript. All authors contributed to the article and approved the submitted version.

## Funding

This study was supported by a grant from the National Natural Science Foundation of China (No. 81871958), a grant from the Shanghai Municipal Health Commission (No. 2020JZX0206), a grant from the Xuhui District Project Specialized for Artificial Intelligence Medical Hospital-District Cooperation of Shanghai (No. 2020-008), and a grant from the Shanghai Science and Technology Innovation Action Plan (No. 19DZ2251800).

## Conflict of Interest

The authors declare that the research was conducted in the absence of any commercial or financial relationships that could be construed as a potential conflict of interest.

## Publisher’s Note

All claims expressed in this article are solely those of the authors and do not necessarily represent those of their affiliated organizations, or those of the publisher, the editors and the reviewers. Any product that may be evaluated in this article, or claim that may be made by its manufacturer, is not guaranteed or endorsed by the publisher.

## Abbreviations

ACS, American Cancer Society; BMI, body mass index; CI, confidence interval; CRC, colorectal cancer; CRA, colorectal adenomas; FIT, fecal immunochemical testing; OR, odds ratio; RCS, restricted cubic spline; SD, standard deviation.
